# Supplementary material for: A Pectate Lyase Gene Plays a Critical Role in Xylem Vascular Development in Arabidopsis
Source: Int J Mol Sci. 2023 Jun 29;24(13):10883. doi: 10.3390/ijms241310883 (PMC10341884; doi:10.3390/ijms241310883)
Supplement: Supplementary file 1 [file ijms-24-10883-s001.zip › Supplementary Figure S1.pdf]

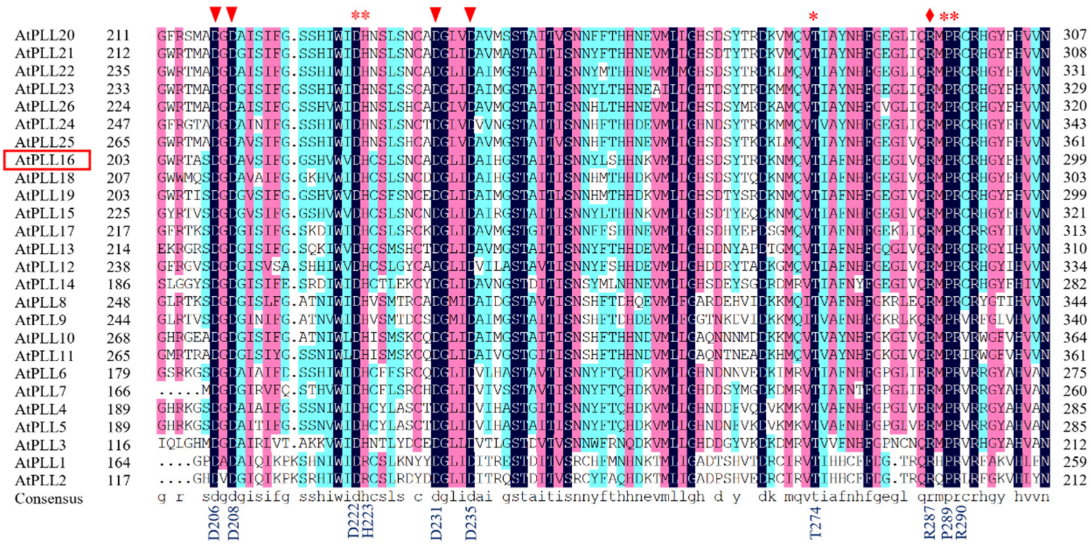

Supplementary Figure S1. Multi-sequence alignment of 26 AtPLL proteins. AtPLL16 is highlighted with a red box, conserved residues for Ca^{++} binding were shown with red triangles, residues involved in substrate binding were shown with red asterisk and catalysis residues was shown with diamond.
