# Supplementary material for: A Pectate Lyase Gene Plays a Critical Role in Xylem Vascular Development in Arabidopsis
Source: Int J Mol Sci. 2023 Jun 29;24(13):10883. doi: 10.3390/ijms241310883 (PMC10341884; doi:10.3390/ijms241310883)
Supplement: Supplementary file 1 [file ijms-24-10883-s001.zip › Supplementary Figure S2.pdf]

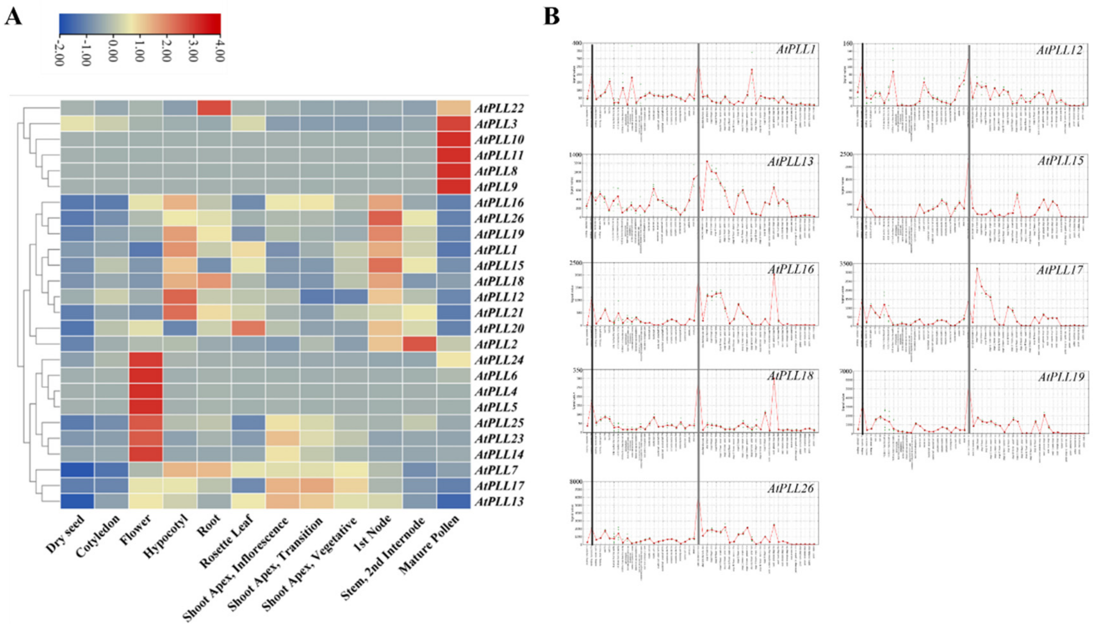

Supplementary Figure S2. Expression analysis of *AtPLLs*. (A) Expression data from Arabidopsis eFP browser. Values across different tissues were horizontally normalized before generation of heat map. HCL clustering between genes were performed and gene groups indicated on the left. (B) *AtPLL* genes preferentially expressed in vascular tissues. Expression data from Aranet website, left black lines represent hypocotyl of *A.thaliana* and right gray lines represent 1st internodes.
