# Supplementary material for: A Pectate Lyase Gene Plays a Critical Role in Xylem Vascular Development in Arabidopsis
Source: Int J Mol Sci. 2023 Jun 29;24(13):10883. doi: 10.3390/ijms241310883 (PMC10341884; doi:10.3390/ijms241310883)
Supplement: Supplementary file 1 [file ijms-24-10883-s001.zip › Supplementary Figure S3.pdf]

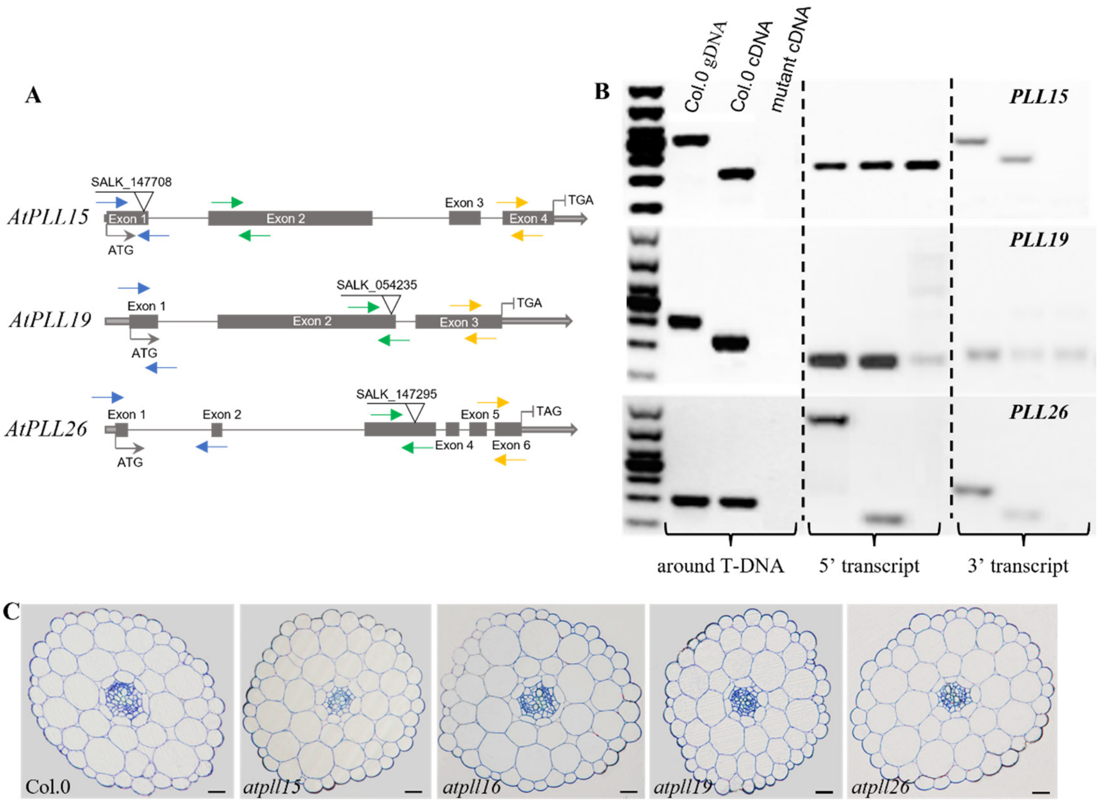

Supplementary Figure S3. Characterization of T-DNA mutants of *AtPLL15*, *16*, *19* and *26*. **(A)** The illustration for T-DNA insertion. The triangle shows the insertion position and the lines on triangles represent insertion direction. **(B)** RT-PCR results for *AtPLL15*, *19* and *26* genes. Dashed line separated results for the three pairs of primers, one spanning the T-DNA insertion site, second corresponding to the 5'-part of transcript, third to the 3'-part of the transcript.Col.0 gDNA and Col.0 cDNA were used as control, top to bottom panels indicated four *AtPLL* genes. DNA ladder was shown on the left. **(C)** Hypocotyl cross section at seedling stage. Scale bar, 20 µm.
